# Opportunities and challenges for integrating the development of sustainable polymer materials within an international circular (bio)economy concept

**DOI:** 10.1557/s43581-021-00015-7

**Published:** 2022-02-09

**Authors:** Natalia A. Tarazona, Rainhard Machatschek, Jennifer Balcucho, Jinneth Lorena Castro-Mayorga, Juan F. Saldarriaga, Andreas Lendlein

**Affiliations:** 1 Institute of Active Polymers, Helmholtz-Zentrum Hereon, Kantstraße 55, 14513 Teltow, Germany; 2 Institute of Chemistry, University of Potsdam, Karl-Liebknecht-Straße 24-25, 14469 Potsdam, Germany; 3 Department of Bioproducts, Corporación Colombiana de Investigación Agropecuaria (Agrosavia), 250047 Mosquera-Cundinamarca, Colombia; 4 Civil and Environmental Engineering Department, Universidad de los Andes (UniAndes), 111711 Bogotá, Colombia

**Keywords:** biomaterial, degradable, functional, life cycle assessment, renewable, sustainability

## Abstract

**Highlights:**

The production and consumption of commodity polymers have been an indispensable part of the development of our modern society. Owing to their adjustable properties and variety of functions, polymer-based materials will continue playing important roles in achieving the Sustainable Development Goals (SDG)s, defined by the United Nations, in key areas such as healthcare, transport, food preservation, construction, electronics, and water management. Considering the serious environmental crisis, generated by increasing consumption of plastics, leading-edge polymers need to incorporate two types of functions: Those that directly arise from the demands of the application (e.g. selective gas and liquid permeation, actuation or charge transport) and those that enable minimization of environmental harm, e.g., through prolongation of the functional lifetime, minimization of material usage, or through predictable disintegration into non-toxic fragments. Here, we give examples of how the incorporation of a thoughtful combination of properties/functions can enhance the sustainability of plastics ranging from material design to waste management. We focus on tools to measure and reduce the negative impacts of plastics on the environment throughout their life cycle, the use of renewable sources for their synthesis, the design of biodegradable and/or recyclable materials, and the use of biotechnological strategies for enzymatic recycling of plastics that fits into a circular bioeconomy. Finally, we discuss future applications for sustainable plastics with the aim to achieve the SDGs through international cooperation.

**Abstract:**

Leading-edge polymer-based materials for consumer and advanced applications are necessary to achieve sustainable development at a global scale. It is essential to understand how sustainability can be incorporated in these materials via green chemistry, the integration of bio-based building blocks from biorefineries, circular bioeconomy strategies, and combined smart and functional capabilities.

**Graphic abstract:**

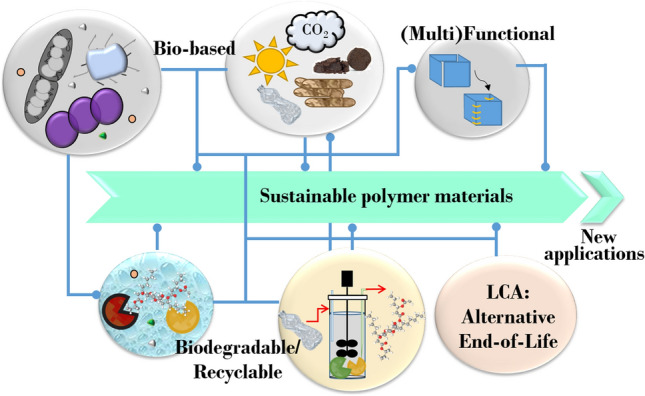

## Discussion

- A new generation of plastics is needed to address the Sustainable Development Goals (SDG)s.
- Data for the End of Life of polymers must be included in their Life Cycle Assessment, and should be supported by predictive methods for degradation analysis.
- Sustainable development takes advantage of renewable resources for the production of materials and of biocatalysts for recycling processes.
- International cooperation should be enhanced in sustainability aspects of polymer research.

## Introduction

Humanity faces several daunting challenges that endanger an acceptable quality of life for future generations, including a growing world population, an increase and acceleration of population aging, the over-use of resources and the increasing risk of conflict for natural resources, climate change and the ineffectual responses to mitigate its effects, among others. The overall goal of sustainable development, defined by the Brundtland Commission, is the long-term stability of the economy and environment, through the integration and acknowledgment of economic, environmental, and social concerns for decision-making processes.^1^ Over the years, three interconnected pillars for sustainable development, namely economic development, social development, and environmental protection, have emerged^2^; yet “sustainability” remains an open concept with countless interpretations based on specific contexts. An important milestone in international sustainability policy was made in 2015 by the United Nations, with the establishment of the 2030 Agenda for Sustainable Development “Transforming our world”, which introduced 17 Sustainable Development Goals (SDGs) in thematic issues including water, energy, climate, oceans, urbanization, transport, science and technology.

Based on report maps of the latest sustainability research and initiatives within each SDG area, materials science contributes to achieving a multitude of goals: Zero hunger (SDG 2), good health and well-being (SDG3), clean water and sanitation (SDG 6), affordable and clean energy (SDG 7), industry, innovation and infrastructure (SDG 9), sustainable cities and communities (SDG 11), responsible consumption and production (SDG 12), climate action (SDG 13), and life below water (SDG 14).^3^ The plastic industry and plastics as ubiquitous and versatile materials are important contributors to most of the above-mentioned goals. However, persistent plastics accumulated in nature are considered as one of the largest environmental threats faced by humans and animals globally. The next generation of polymers that contribute to the SDGs therefore has to be redesigned to fit into a more resource-efficient and circular economy. The plastic problem originates partially from an increase of plastic waste due to a linear economy that follows a “take-make-dispose” scheme,^4^ where non-renewable materials are used to make products that are frequently disposed of at the end of their short useful life; together with waste management systems that often do not prevent leakage from landfill sites. Recycling is one of the solutions implemented to address the problem. Nonetheless, from the total amount of plastic waste “ever” generated worldwide between 1950 and 2015 (thermoplastics, thermosets, elastomers, and synthetic fibers, commonly referred to as “plastics”), only about 9% have been recycled, while 12% have been combusted with energy recovery and 79% were discarded to landfills or in the natural environment, which accounts for about 60% of all plastics ever produced.^5^ Since 2017, increasing amounts of plastics must be processed or recycled domestically due to the ban on the import of plastics by the world´s largest importer, China.^6^ This increases the importance of landfilling as the first option for the disposal of post-consumer plastic waste in many European countries.^7^ Besides the accumulation in landfills, plastic waste carried by waterbodies prompts negative effects on several terrestrial and aquatic ecosystems, since most disposed commodity plastics (produced in high volume) such as polyethylene terephthalate (PET), high, low, and linear-low density polyethylene (HDPE, LDPE, and LLDPE), polyvinyl chloride (PVC), polypropylene (PP), and polystyrene (PS), are chemically stable for decades. For instance, a plastic bottle made of HDPE with an approximate wall-thickness of 500 µm will take 250 years to lose the first 50% of the polymer mass (half-lives) when buried in land or 58 years to do the same in a marine environment.^8^

Alternatives for alleviating the plastic crisis include the implementation of extended producer responsibility; the reduction and recycling of single-use plastics using strategies beyond the traditional recycling via melting and re-extrusion^9^; the development of more “sustainable” plastics, in which an evaluation of their impacts through their lifecycle is included.^10, 11^ In this perspective, we address the challenges and strategies for the development and incorporation of sustainable plastics (including bio-based, biodegradable materials, and those containing both features) within the frameworks of Sustainable Green Chemical Principles (GCP),^12^ circular economy (CE)^12^, and bioeconomy. We discuss life-cycle assessment, sustainability-by-design, and biorefineries as strategies to increase sustainability in polymer research and development (“Strategies to increase sustainability in polymer science” section); we offer some insights into future applications of these polymers toward achieving the SDGs (“Sustainable polymers to address the SDGs” section); and explain the importance of integrating industry, academia, and governments to ensure effective implementation of sustainability practices in polymer science (“International cooperation for sustainable polymer materials” section).

## Strategies to increase sustainability in polymer science

**Figure 1:**
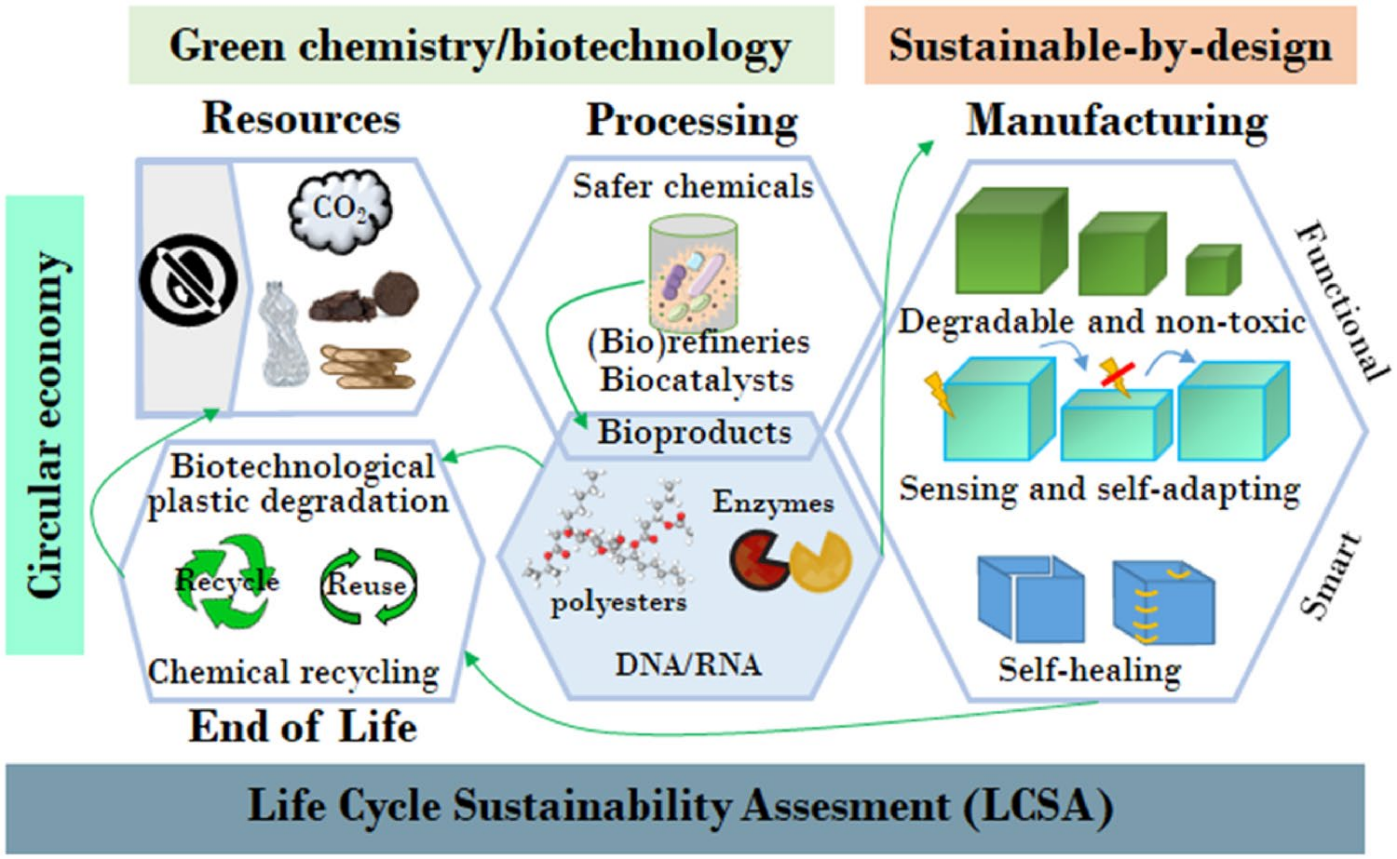
Contributions of polymer science and biotechnology for the sustainable development of plastics. Further to the traditional circular economy concept and the integration of LCSA, the development of polymer-based materials should adopt green chemistry principles (less hazardous chemical synthesis, design of safer chemicals, using renewable feedstock, design for degradation, etc.) and biotechnology (biocatalysts, multi-product biorefinery, and bioproducts that can be directly incorporated into materials). Moreover, the design of smart and functional materials is envisioned to enhance reuse, recycling, and degradation strategies.

When listing the benefits of plastics to our daily life, it is indispensable to point out the challenges arising from the production of these materials, which generally exceeds the current capacities to transfer them into a closed loop with minimal waste generation and energy loss. While a CE aims to improve resource and energy efficiency by keeping the resources circulating for as long as possible, through efficient materials use, reuse, and recycling loops, a circular bioeconomy (CBE) integrates both a CE and a production based on renewable biological resources.^4, 13–15^ From our perspective, a true transition toward sustainable polymers has to attain a holistic approach that includes: (a) accountable methodologies to determine, predict, and reduce the impact of polymers through their life cycle; (b) the incorporation of GCP, CE, and sustainability-by-design principles in the development of new polymers; and (c) the development of refineries for bioconversion of second-generation biomass (non-edible biomass and plastic waste) into biomolecules (polymers and enzymes) for recycling and degradation of plastics (Fig. 1).

### Life cycle analysis

Procedures and methods of Life Cycle Assessment (LCA), consolidated in the international standards for LCA (ISO series 14040-14044), were introduced as a systematic tool to address the potential environmental impacts throughout the entire life cycle of a product, from raw material to use and end of life (EoL) treatment and final disposal.^16^ Although the economic and social aspects are outside of the scope of traditional LCA, it has become a widespread decision-support tool bringing together policy-makers, industry, researchers, and other stakeholders in assessing the cradle-to-grave impacts of a product or process, allowing a sensible material selection. Moreover, with life cycle sustainability assessment (LCSA), a comprehensive tool, that includes sustainability assessment in all its dimensions, is under development.^17^

LCA has produced extensive studies, where bio-based and fossil-based polymers are compared, especially in food packaging.^18^ As a general outcome, a positive correlation between adherence to green design principles -the “12 GCP” and the “12 Principles of Green Engineering”- and the reduction of the impacts of fossil-based and bio-based polymers has been demonstrated.^19^ In the focus of this perspective, we would like to highlight the LCA findings by Gerassimidou *et al.*^20^ In this study, an integrated sustainability matrix was developed to depict the challenges and trade-offs of introducing bio-based plastics (degradable and non-degradable) in the food packaging value chain. Higher environmental and social impacts, when compared to conventional plastics, were identified due to: (i) cultivation of 1st generation feedstock (namely sugar cane, corn, wheat, cassava) together with fertilizer use, pesticide use and exposure of farmers and land-use change required for agricultural production; (ii) infrastructure demand and operational costs required for establishing a biorefinery process; and (iii) technology associated problems with mechanical recyclability. On the other hand, positive impacts for the introduction of bio-based polymers in packaging were found in the expansion of agricultural economies, which could create more income for rural communities, while novel biorefinery concepts could create added value by including different products and optimizing processes.^20^

In this subject, one of the biggest challenges of comparative studies using LCA is incorporating scientific data for the EoL of polymers that are not yet on the market, as well as for alternative biological or chemical recycling and synthesis methods using second-generation biomass. In the case of polymers for single-use applications such as packaging, this will result in the EoL fate exerting a considerable influence on sustainability and therefore should be included in LCA studies that compare bio-based and biodegradable polymers with petrochemical benchmarks. It is foreseeable that predictive models that simulate the fate of (new) plastics produced from waste or biomass will play key roles in future CE and LCA.

### Development of sustainable-by-design polymers for a sustainable circular (bio)economy

In view of sustainable development, polymer scientists will need to focus on improving the overall circularity and sustainability of the materials they develop. For instance by extending their lifespan, decreasing degradation times, decreasing material use, using renewable feedstock (1st or 2nd generation), enhancing separability of components and recyclability (Fig. 1). Two important criteria for current sustainable polymers are the use of renewable feedstock and the degradability or recyclability of the polymer,^10^ which combined are the key to a CBE. Bio-based plastics can be roughly divided into two classes: (i) degradable polymers such bio-based polybutylene succinate (Bio-PBS), polylactides (PLA), and polyhydroxyalkanoates (PHAs); and (ii) slowly degrading polymers, structurally identical or very similar to fossil-based plastics, like Bio-PET, Bio-PE (Bio-polyethylene), and bio-sourced nylon. On the other hand, degradable plastics can also have a petrochemical origin, e.g., poly[(butylene adipate)-co-terephthalate] (PBAT) and PBS; however, we will not address those in this perspective article. Bio-based polymers for which fossil-based counterparts play no role, e.g. poly(ethylene-2,5-furanoate) (PEF), have been recently brought into the spotlight. PEF is produced via polycondensation of 2,5-furandicarboxylic acid (FDCA), derived from C6 sugars, in presence of ethylene glycol. This polymer has the potential to replace PET and polybutylene terephthalate (PBT) in many applications, due to superior barrier performance as well as mechanical and thermal properties compared to other bioplastics.^21, 22^ The furanics family is a clear example of bio-based plastics with huge market potential, to the extent that they are referred to as “sleeping giants” of renewable intermediate chemicals for the synthesis of polyesters, polyamides, and their copolymers.^21^ Still, careful consideration of the environmental and economic impact of renewable sources for the production of bio-based polymers is imperative to prevent their mass production from adding to the global competition for local natural resources.^23^

Biodegradable polymers derived from biological sources, such as PLA and PHAs, have been studied and suggested for short-term products such as packaging, foils, and utilization in agriculture, especially where emissions and leakage into the environments are intended or inevitable. Several excellent reviews on degradable polymers have been published in the past few years, focusing on their challenges and opportunities, including slow degradation rates (as in the case of PLA in marine environments) that could cause an excess of wastes and associated environmental problems.^22, 24–26^ Innovations are necessary to develop a more cyclic model of resource utilization for bio-based and biodegradable polymers at the end of their useful life. We see potential for three specific strategies being currently explored mostly in traditional polymers. The first one is to design backbone-degradable polymers with improved and selective depolymerization that allow both chemical degradation to their constituent monomers or other useful intermediates and degradation in the environment by microorganisms. Several concepts have been investigated, including the introduction of chemically labile groups (such as esters, carbonates, amides, or acetals) and the introduction of stimuli-responsive motifs, which induce degradation of polymers by temperature or light-induced cleavage. Adding to this, one should keep in mind the polymers’ biodegradability potential, predicted as polyesters > polyamides > polyolefins.^27^ The second strategy is to implement (multi)functionality into polymer-based materials to enhance their resilience and to extend their lifespan increasing the opportunities for recycling and reuse. Among those functions, self-healing could provide materials with the capacity to modify their structure for repair and to retain a specific function in case of potential damage.^28–30^ More ambitious functions in polymer-based materials involve sensing, self-adapting (in case of irreversible damage), and on-demand dismantling (in multi-material products), which will allow materials to adjust to changes in the environment and reduce the need for replacement. The third option is to upgrade polymer-degrading enzymes, which can be used for efficient recycling of polymer waste, and as bioremediatiors to accelerate degradation.^27^

### Biotechnological approaches in bioeconomy

As part of the bioeconomy concept, biorefinery approaches that provide alternatives to established forms of production and consumption patterns are at the vanguard in the fields of biotechnology and polymer science. A biorefinery, i.e. a refinery that converts biomass to energy and other beneficial byproducts, is based on four principles: sustainability, cascading (as many products as possible), non-conflict with food, and neutral carbon footprint.^31^ The most common biorefinery concept deals with the synthesis of bioproducts from lignocellulose, including bioenergy,^32^ biofuels,^31^ and valuable building blocks like lactic acid (LA), isosorbide (IS), FDCA, p‐xylene (pXL)^33^ and more recently, non-isocyanate polyurethane foams.^34^ Microorganisms and their enzyme machinery can carry out nearly all steps from lignocellulose pretreatment to lignocellulose hydrolysis and fermentation. Therefore, it is necessary to develop resilient and efficient production organisms for biorefineries (plants, algae, fungi and bacteria), while expanding the product range capacity.

In polymer science, enzymes have become interesting micromachines for several reasons: (i) enzymes can be used to synthesize plastics through a 100% biotechnological approach, e.g. PHAs,^35, 36^ (ii) polymer-degrading enzymes have been discovered for both degradable polymers and materials formerly considered as “recalcitrant polymers” such as PET.^37, 38^ PHAs are prominent examples of the potential of microorganisms to produce bio-based and biodegradable polymers, whose properties are tunable by varying the side-chain of the repeating units. This structural variation does not only influence the thermomechanical properties of the material,^39^ but also its degradation behavior,^40^ offering multiple strategies for EoL management. Although PHAs can be degraded in soils by numerous microorganisms, microbial degradation still depends on the polymer's chemical structure, molecular weight, and crystallinity, needing improvements also at the polymer synthesis level.^40–42^

In the latest years, scientists have identified and engineered enzymes to meet requirements for efficient depolymerization and recycling of recalcitrant polymers such as PET and polyurethanes.^37, 38^ A recent study presented an enhanced PET hydrolase that ultimately achieves, over 10 h, a minimum of 90% PET depolymerization into monomers.^43^ These results are promising considering the novelty of the strategies for biotechnological degradation and recycling of plastics. This is a clear invitation for more research and development of new organisms that can extend the portfolio of classic biotechnology.

## Sustainable polymers to address the SDGs

Due to the ubiquity of polymers in everyday life, one cannot deny the positive contributions of polymers to the three sustainable development pillars. In this section, we will focus on specific examples where polymers make a tangible positive contribution to the SDGs: membranes for water filtration (SDG 6); shelf life extension of fresh fruits, vegetables, and animal-based proteins for safe food (SDG 2, SDG 3); and materials for medical applications (SDG 3). Although food packaging remains a significant application field of bio-based and biodegradable polymers, we highlight the increasing trend toward using more sustainable polymers to bring new functional attributes for specialized and commodity applications with high-value markets (antimicrobial function, solvent barrier and chemical protection, electrical conductivity, etc.). The introduction of such high-value materials will allow early actions to ascertain their compatibility with the CBE framework, as well as an expansion in the production capacity of bio-based and degradable polymers.

Membrane-based separation systems have been used to tackle water scarcity and the pollution of aquatic environments, due to their capacity to reduce salinity, remove particles and microbial pathogens, reduce natural organic matter and to remove dissolved toxic metals and oily compounds.^44–46^ Polymer membranes, including phase inversion membranes, thin-film composite polyamide membranes and self-assembled membranes provide exciting opportunities as high-performance membranes for microfiltration, ultrafiltration, nanofiltration (NF), reverse osmosis (RO) and forward osmosis processes. The increasing environmental restrictions imposed for polyamide-based RO and NF membranes, which dominate the desalination market,^46^ are pushing the transition toward greener alternatives, which include following the GCP, and the use of bio-based polymers. For instance, cellulose acetate-based membranes have been used extensively at an industrial scale to reduce the microorganism content in raw waste and for e.g. RO processes.^47^ Current research in membrane science is pointing toward bio-based polymers (cellulose, alginate, chitosan, Bio-PBS, PLA, among others), their preparation via phase inversion and polymer extrusion, and strategies to overcome challenges such as limited mechanical properties using nanoparticles, natural plasticizers, and polymer blends.^46, 47^

Moreover, polymers are certainly an important material in food packaging required for food safety and the protection of consumer health. Current bio-based and degradable polymers used in the food sector are PLA (examples are bowls, bags and yogurt jars), starch-based (e.g. cornstarch trays for chocolates), and cellulose-based materials (such as trays and metalized cellulose film for sweets and potato chips).^48^ A nascent field of packaging is the development of active packaging materials, which include advanced materials with improved properties (barrier properties, mechanical strength, heat resistance and anti-microbial activity) and sensors for the detection of food contaminants or monitoring the packaging conditions integrity.^49^ Bioactive agents, like antimicrobial metal nanoparticles, essential oils, encapsulated tannins, vitamins, and enzymes, can be integrated into polymer films or directly on the surface of the food as thin edible multilayer coating. While it is clear that expanding the current applicability of bio-based and biodegradable polymers for traditional food packaging and active packaging requires additional efforts, emerging areas of research such as nanoscale reinforcements of plastic packaging with nanoparticles, thin films, nanowires, and bulk materials made of nanoscale building blocks or nanoscale structures, are opening new horizons in the application field.^50^

In modern medicine, polymer-based materials are applied in single-use items like sterile packaging, syringes, drip bags, catheters, etc. They are indispensable for orthopedic and surgical devices, as they are suitable materials for the production of flexible implants. With a simultaneously growing and aging world population and steadily enhancing medical standards in developing countries, the demand for plastics for health can only grow. Polymer-based materials are also the foundation of many developing fields and techniques in medicine such as controlled drug release, regenerative medicine, minimally invasive surgery for implants of biomedical devices, and precision (or personalized) medicine using additive manufacturing of implants and orthopedics.^51^ While the capability of plastics to be processed into nearly any shape by low-cost, high throughput production methods can be regarded as one of the root causes of their careless consumption, the COVID-19 emergency, declared a pandemic by the World Health Organization (WHO), has demonstrated impressively how the same properties constitute an invaluable benefit. Within weeks, the global production of respiratory protection devices has been increased to meet the sudden demand of every human on the globe. In this way, polymer materials have become the first line of defense against the virus and its further spread. Air filtering masks are produced from polymers and consist of three polymeric layers; an inner layer (soft fibers), a middle layer (melt-blown and main filter), and an outer layer (nonwoven fibers).^52^ The most essential component of a mask is the middle filtration layer, also known as the fibrous medium, which is based on melt-blown PP, a polyolefin thermoplastic polymer. With the knowledge of bio-based and degradable materials and the performance needs for biomaterials as consumer products, current strategies for more sustainable production of masks are focusing on biodegradable polymers, such as cellulose, starch, chitin and chitosan as alternatives to the filter medium.^52^

## International cooperation for sustainable polymer materials

As demonstrated by the COVID-19 Pandemic, overcoming a global emergency requires solutions that are implemented and adapted locally, but on a worldwide scale. The SGD 17 (Partnership for the Goals) is the meta-goal to mobilize and share knowledge, expertise, technologies and financial resources (at the global, regional, national and local levels) to support the achievement of the goals in all countries, particularly developing ones. Some of the nineteen targets of SDG 17 bring science for sustainable development into an international context.

The progress toward these targets can be measured by the number of science and/or technology cooperation agreements and programs between countries, by type of cooperation (Target 17.6); and the total amount of approved funding for developing countries to promote the development, transfer, dissemination, and diffusion of environmentally sound technologies (Target 17.7). A bibliometric overview of the SDGs in the scientific literature at the meta-level suggests that researchers are generally on a good track concerning international collaboration, since nearly 37% of all articles dealing with SDGs count as international publications, i.e., as being co-authored by authors from affiliations of multiple countries. Moreover, Life Sciences and Biomedicine were regarded as the most prevalent research area in the dataset.^53^

The EU has made it a high priority to use biorefinery concepts to turn biomass into a valuable resource, through several funding programs for international cooperation.^54^ The concept of bioeconomy has gained importance in Latin America and the Caribbean, given that the continent’s mega biodiversity offers great potential for biomass production and utilization (Report on Bioeconomy Policy (Part III): Update Report of National Strategies around the World from the German Bioeconomy Council, 2018).^55^ An example is the Bioeconomy International program from the German Federal Government and its National Bioeconomy Strategy, which is aiming at solutions for both waste disposal and creating value-added products. Within the framework of international cooperation in bioeconomy, researchers and other stakeholders should develop operative approaches that fit into the safe, sustainable, and cost-effective development of polymers as materials and enzyme biocatalysts with biotechnological potential at an industrial scale, while contributing to tackling challenges in environmental research.

## Conclusion and outlook

In the times where phrases like "be sustainable", "do sustainable", "make sustainable", and "use sustainable" abound in our day-to-day conversations, it is important to not elude the responsibility to conserve our planet for further generations. Scientists and lawmakers alone cannot push all buttons required to transform our current way of living and using our resources. Besides the numerous strategies mentioned in this perspective to deal with the specific challenges of resources depletion and plastic pollution, sustainable science and sustainable materials could benefit from existing and emerging technologies, for instance, digitalization and artificial intelligence. The latter refers to any software with at least one of these capabilities: perception, prediction, automatic knowledge extraction and recognition of data, interactive communication, or logical reasoning. It is undoubtful that its capacities especially in analyzing and extrapolating from huge datasets will help to better understand both the scale and the causes of the global plastics crisis and support the development of new strategies against it, as it has been attractively exemplified recently.^27^ Yet, addressing a problem of global scale with data-driven approaches requires sufficient and unbiased data from every region of the world, which again highlights the need for international collaboration. The growth of science clusters is also envisioned to facilitate knowledge and infrastructure sharing and to avoid redundant research to accelerate the transformation. Here, especially increasing accessibility of scientific research, e.g., through open access publishing and sharing original data through repositories, are in the spotlight. Finally, when considering international collaboration projects, it is mandatory to follow the guidelines for sustainable partnership, to ensure that no nation or ecosystem is harmed.

## Data Availability

The manuscript has no associated data.
